# Promoter hypomethylation and overexpression of TSTD1 mediate poor treatment response in breast cancer

**DOI:** 10.3389/fonc.2022.1004261

**Published:** 2022-11-07

**Authors:** Muhamad Ansar, Le Thi Anh Thu, Chin-Sheng Hung, Chih-Ming Su, Man-Hsu Huang, Li-Min Liao, Yu-Mei Chung, Ruo-Kai Lin

**Affiliations:** ^1^ Ph.D Program in the Clinical Drug Development of Herbal Medicine, Taipei Medical University, Taipei, Taiwan; ^2^ Graduate Institute of Pharmacognosy, College of Pharmacy, Taipei Medical University, Taipei, Taiwan; ^3^ Quang Tri Medical College, Dong Ha, Quang Tri, Vietnam; ^4^ Division of Breast Surgery, Department of Surgery, Taipei Medical University Hospital, Taipei, Taiwan; ^5^ Department of Surgery, School of Medicine, College of Medicine, Taipei Medical University, Taipei, Taiwan; ^6^ Division of General Surgery, Department of Surgery, Shuang Ho Hospital, Taipei Medical University, New Taipei City, Taiwan; ^7^ Department of Pathology, Shuang-Ho Hospital, Taipei Medical University, New Taipei City, Taiwan; ^8^ Master Program in Clinical Genomics and Proteomics; Ph.D. Program in Drug Discovery and Development Industry, College of Pharmacy, Taipei Medical University, Taipei, Taiwan; ^9^ Clinical Trial Center, Taipei Medical University Hospital, Taipei, Taiwan

**Keywords:** *TSTD1*, breast cancer, DNA methylation, hypomethylation, chemotherapy, hormone therapy, drug response, circulating cell-free DNA (cfDNA)

## Abstract

Epigenetic alterations play a pivotal role in cancer treatment outcomes. Using the methylation array data and The Cancer Genome Atlas (TCGA) dataset, we observed the hypomethylation and upregulation of thiosulfate sulfurtransferase–like domain containing 1 (*TSTD1*) in patients with breast cancer. We examined paired tissues from Taiwanese patients and observed that 65.09% and 68.25% of patients exhibited *TSTD1* hypomethylation and overexpression, respectively. A significant correlation was found between *TSTD1* hypomethylation and overexpression in Taiwanese (74.2%, *p = 0.040*) and Western (88.0%, *p < 0.001*) cohorts. High expression of TSTD1 protein was observed in 68.8% of Taiwanese and Korean breast cancer patients. Overexpression of *TSTD1* in tumors of breast cancer patients was significantly associated with poor 5-year overall survival (*p* = 0.021) and poor chemotherapy response (*p* = 0.008). T47D cells treated with *TSTD1* siRNA exhibited lower proliferation than the control group, and transfection of *TSTD1* in MDA-MB-231 induced the growth of MDA-MB-231 cells compared to the vector control. Additionally, overexpression of *TSTD1* in MCF7 cells mediated a poor response to chemotherapy by epirubicin (*p* < 0.001) and docetaxel (*p* < 0.001) and hormone therapy by tamoxifen (*p* =0.025). Circulating cell-free hypomethylated *TSTD1* was detected in plasma of Taiwanese breast cancer patients with disease progression and poor chemotherapy efficacy. Our results indicate that promoter hypomethylation and overexpression of *TSTD1* in patients with breast cancer are potential biomarkers for poor 5-year overall survival and poor treatment response.

## Introduction

Breast cancer is the leading cause of cancer mortality and Breast cancer is the leading cause of cancer mortality and morbidity in women worldwide, followed by lung cancer (1). Despite the development of several treatment strategies, the mortality rate of patients with breast and lung carcinoma is considerably high: 30–35 per 100 people diagnosed with either cancer die (1, 2). Delaying the diagnosis leads to a significant decline in the survival rate of patients. The 10- year survival rate drops by half when patients proceeded to Stage III (3). Only 22% of stage IV breast cancer patients will survive for a subsequent 5 years (4). The likelihood of patients with Triple Negative Breast Cancer (TNBC) surviving after 5 years decreases to approximately 20% compared to patients without TNBC (5). Accordingly, researchers have been making substantial efforts to improve the diagnostic modalities and treatment outcomes for breast cancer (6–10). Biomarker discovery is a promising approach to reduce cancer mortality (11, 12). Identifying a tumor marker that differentiates between cancerous and noncancerous tissues is crucial.

Four intrinsic molecular subtypes of breast cancer (Luminal A, Luminal B, HER2-enriched [HER2-E] and Basal-like) have been identified (13, 14). Chemotherapy and hormonal therapy are widely used to treat patients diagnosed with Luminal A and Luminal B breast cancer and can prevent recurrence (15, 16). Chemotherapy was also reported to benefit patients with potentially malignant luminal A breast cancer (17). The most commonly used single-agent cytotoxic drug classes used in chemotherapy include taxanes (docetaxel, paclitaxel, nab-paclitaxel), anthracyclines (doxorubicin, epirubicin, pegylated liposomal doxorubicin) (18, 19). In more than 50% of patients, chemotherapy treatment fails due to sensitivity and resistance (20, 21). Tamoxifen is one of the most commonly used hormone therapies for breast cancer. However, hormonal therapy resistance was apparent in 30-40% cases (22, 23), and takes a long time to complete (24). TNBC has a poor prognosis, largely due to the lack of targeted treatments. The high response rates to chemotherapy are lack longevity due to the early development of resistance mechanisms (25).” Personalized medicine might provide more favorable outcomes of cancer treatment. Several irregularities in an individual’s gene expression are associated with chemotherapy outcomes (26). For instance, miRNA-631 expression increased paclitaxel response in breast cancer patients (27), miR-145-5p downregulation was found to be correlated with Paclitaxel resistance in MCF-7 and MDA-MB-231 cell lines (28), and overexpression of SH3BGRL conferred resistance to cisplatin (29). Researchers are attempting to identify a novel diagnostic biomarker that can serve as the fundamental building block for cancer treatment strategies.

In the current study, we first determined methylation patterns of tissue by analyzing data from The Cancer Genome Atlas (TCGA) dataset. We identified thiosulfate sulfurtransferate-like domain containing 1 (*TSTD1*) as a potential biomarker specific to breast cancer. *TSTD1*, located on chromosome 1q23.3 and expressed in the cytoplasm close to the nuclear membrane, has been proposed to play an essential role in sulfur-containing group transfer and cyanide detoxification (30–33). Unlike other sulfurtransferases—mitochondrial rhodanese and mercaptopyruvate sulfurtransferase—whose functions in sulfide biogenesis and oxidation pathways have been relatively well-described (34, 35), the physiologically relevant reaction catalyzed by *TSTD1* remains unclear. Studies have reported that *TSTD1* acts as a catalyst in the mitochondrial sulfide oxidation pathway, producing glutathione persulfide (GSS^−^) from glutathione (GS^−^) (31), and plays a crucial role in sulfide-based signaling (30). Regarding expression, TSTD1 protein levels were not detected in normal breast cell lines (MCF-10A and H184A1) but were high in breast cancer cell lines (30, 32). However, no comprehensive study has evaluated underlying alterations in *TSTD1* in cancer and its applications in clinical practice. This study investigated changes in *TSTD1* methylation and expression in patients with breast cancer compared to normal tissues to determine the potential of *TSTD1* as a tumor biomarker in clinical practice in breast cancer.

## Methods

### Candidate gene selection using TCGA

The results for Western patients, including the methylation status, mRNA expression level, and clinical information, were analyses of data provided by TCGA Research Network (http://cancergenome.nih.gov/). TCGA contains information regarding critical cancer-related genome aberrations, covering all levels of DNA, RNA, miRNA, and proteins in cohorts of more than 83,000 cases with 33 cancer types, including 8897 breast cancer samples (36, 37).

### Patient Enrolment and tissue collection

To analyze whether the alterations of *TSTD1* is specific to breast cancer patients, paired tumor and noncancer tissues from 106 patients with breast cancer, 33 patients with lung cancer, 15 patients with endometrial cancer, and 16 patients with esophageal cancer were collected from the Taipei Medical University Joint Biobank. This study was approved by the Joint Institutional Review Board of Taipei Medical University and the Institutional Review Board of Taipei Medical University-Shuang Ho Hospital, Ministry of Health and Welfare. All patients provided written informed consent. Clinical data regarding age, race, personal and family medical history, tumor location, tumor–node–metastasis (TNM) stage (38), tumor classification (39), and follow-up conditions were prospectively collected. Breast cancer patients were followed up for at least 15 months. According to the expression levels of Ki-67 protein and the status of estrogen receptor (ER), progesterone receptor and human epidermal growth factor receptor 2 (HER2), breast cancer can be categorized into four subtypes: luminal A, luminal B, HER2-overexpression, and triple negative breast cancer (TNBC) (40). A senior pathologist examined partial specimens of cancerous and adjacent normal tissues. Patient survival time was investigated following treatments, patients were monitored every 3 months for the first 2 years and semi-annually thereafter. The follow-up protocol included physical examination, breast ultrasound examination, carcinoembryonic antigen analysis (CEA), carbohydrate antigen 15-3 (CA15-3), abdominal sonogram, and computerized tomography.

### DNA and mRNA purification from tissues

The matched pairs of cancerous and corresponding noncancerous tissues from the same patient collected during surgery were immediately stored at −80°C and then in liquid nitrogen. Genomic DNA and mRNA were extracted from 106 and 63 tissue pairs respectively using the QIAamp DNA mini kit (Cat. No. 51306, Qiagen, Bonn, Germany) or the RNeasy plus mini kit (Cat. No. 74134, Qiagen), as appropriate. Subsequently, DNA and RNA were quantified, and their purity was determined by measuring the A260–A280 ratio (which ranged from 1.8 to 2.0) (41) using Thermo Scientific NanoDrop 2000c (Thermo Fisher Scientific, MA, USA). Genomic DNA and RNA were stored at −20°C and −80°C, respectively, for subsequent experiments.

### Manual circulating cell-free DNA extraction

Circulating cell-free DNA (cfDNA) from plasma samples was extracted using the MagMAX Cell-Free DNA Isolation Kit according to the manufacturer’s recommended protocol (Thermo Fisher Scientific, Austin, TX, USA). The cfDNA samples had clear fragment size peaks between 140 and 200 bp. The plasma was isolated within 2 hours from 10 mL of peripheral blood within 2 h. After DNA quantification, the purity was verified by measuring the A260/A280 ratio (range 1.8 to 2.0) using a NanoDrop ND-1000 spectrophotometer (NanoDrop Technologies, Inc., Wilmington, DE, USA).

### DNA bisulfite conversion

Bisulfite conversion was conducted using the EpiTect Fast DNA Bisulfite Kit (Cat. No. 59826, Qiagen). A mixture of a maximum of 500 ng of purified DNA, stabilized by DNA protection buffer, and bisulfite solution was incubated in a Labcycler 480 thermal cycler (Sensoquest, Gottingen, Germany). Subsequently, a cleanup procedure was performed in accordance with the manufacturer’s recommended protocol. Bisulfite products were stored at −20°C for future experiments.

### DNA methylation assay

After bisulfite conversion, we determined DNA methylation patterns in paired tumor and normal tissues collected from five Taiwanese patients using Infinium Human Methylation 450K BeadChips (Illumina, San Diego, CA, USA) for one patient and EPIC Methylation Beadchips (Illumina) for four patients. In the DNA methylation assay, beta values were used to score the methylation level. Beta values are the ratio of the intensity of the methylated signal to the overall (sum of methylated and unmethylated) signal and thus ranged from 0 (no methylation) to 1 (full methylation). To design *TSTD1* methylation–specific primers, we used MethPrimer (42) and Methyl Primer Express (v1.0, Thermo Fisher Scientific).

### Quantitative methylation-specific polymerase chain reaction

After bisulfite treatment, the DNA from 106 tissue pairs of patients with breast cancer was used for TaqMan quantitative methylation-specific (qMSP) to measure the DNA methylation level of *TSTD1*. qMSP was performed using the SensiFAST SYBR No-ROX kit (Cat. No. BIO-98020, Bioline, London, UK) or the SensiFAST PROBE No-ROX kit (Cat. No. BIO-86020, Bioline). The assay was measured in a LightCycler 480 (Roche Applied Science, Mannheim, Germany) or LightCycler 96 (Roche Applied Science). Target DNA methylation values were calibrated to the internal control and analyzed using LightCycler Relative Quantification software (version 2.0, Roche Applied Science). Experiments were conducted in triplicate.

The QMSP conditions were as follows: preincubation at 95°C for 10 min followed by 50 cycles of amplification at 95°C for 10 s and 60°C for 10 s. The *beta-actin* (*ACTB*) was used as the reference gene for qMSP. *TSTD1* was considered hypomethylated when the methylation level of *TSTD1* relative to *ACTB* in the breast tumor tissue was no greater than 0.5 times that of the corresponding normal tissue. Bisulfite sequencing was performed to confirm the specificity of *TSTD1* methylation end products (**Figure S1**).

*TSTD1* and *ACTB* methylated–specific primers and probes were designed using MethPrimer (42) and Methyl Primer Express (v1.0, Thermo Fisher Scientific). The primers and probe are described in **Table S1**.

### Quantitative reverse transcription polymerase chain reaction

Reverse transcription PCR (RT-PCR) was performed using FIREScript RT cDNA synthesis mix (Cat. No 06-20-00100, Solis Byodine, Tartu, Estonia). A total of 5 μg of purified mRNA was mixed with the required nucleotides and enzymes provided. The mixture was then placed in Labcycler 48 thermal cycler (Sensoquest, Gottingen, Germany) at the optimal temperature in accordance with the manufacturer’s recommended protocol. Complementary DNA was stored at −20°C for subsequent experiments.

Complementary DNA—the product of reverse transcriptase reaction—was used to determine the mRNA expression level through quantitative RT-PCR (RT-qPCR) in a LightCycler 480 (Roche Applied Science) or LightCycler 96 (Roche Applied Science) system. The PCR conditions were as follows: preincubation at 95°C for 10 min followed by 40 cycles of amplification at 95°C for 10 s and 60°C for 10 s. The glyceraldehyde 3-phosphate dehydrogenase gene (*GAPDH*) was used as an internal control. The LightCycler Probe Master kit (Roche Applied Science), gene-specific primers, and the corresponding probes from Universal ProbeLibrary (Roche Applied Science) at appropriate concentrations were added at appropriate concentrations to the template DNA. The gene specific primers were for *TSTD1*, *GAPDH*, *ESR1*, and *ESR2.* Gene expression values normalized to the internal control were calculated using LightCycler Relative Quantification software (ver. 2.0, Roche Applied Science). *TSTD1* was considered to be upregulated when the expression level of *TSTD1* relative to that of *GAPDH* was at least 1.5 times higher in tumor tissues compared to paired normal breast tissues. Initially, experiments were conducted in triplicate to examine the stabilization of results (n = 40). However, a lack of samples caused experiments to be performed once for each sample (n= 23). The sequences for the primers and probes are listed in **Table S1**.

### Immunohistochemistry assay

*TSTD1* expression was determined by immunohistochemistry in 59 and 104 tissue samples from South Korean and Taiwanese patients with breast cancer, respectively. The samples were preserved as three sets of tissue microarrays. Two of them were obtained from Taipei Medical University-Shuang Ho Hospital, Taiwan, and the other was purchased from SuperBioChips Laboratories (Cat. No.: CBA4, Seoul, South Korea). The pathological diagnoses of these cases were microscopically confirmed by a senior pathologist. An iView DAB detection kit (Ventana, Tucson, AZ, USA) was used for immunohistochemical staining on a BenchMark XT autostainer (VENTANA, Roche Diagnostics, Basel, Switzerland). The sections were incubated with a TSTD1 antibody (1:100, Cat No.: HPA006655, Sigma Aldrich, MO, USA) for 52 minutes at 37°C. This assay included both positive and negative controls. The clinical follow-up data were not provided to the pathologist who evaluated the immunohistochemistry staining. TSTD1 protein expression was classified semiquantitatively as low when it was weaker than or equal to the expression intensity of the normal breast epithelium or high when it was stronger than the expression intensity of the normal breast epithelium.

### Cell culture, siRNA, and transfection

All cells were incubated at 37°C and 5% CO_2_. T47D, MDA-MB-231 and H1299 cell lines were cultured in Dulbecco’s Modified Eagle Medium/Nutrient Mixture *F*-*12* (DMEM/F-12; Gibco, NY, USA) supplemented with 5% human placental lactogen (HPL) serum and 1% penicillin. MCF7 cells were cultured in DMEM/F12 supplemented with human platelet lysate (hPL, American Red Cross, USA), 7.5% HPL, 1% MEM Non-Essential Amino Acid and 1% penicillin.

T47D and H1299 cells were transfected with 10 nM *TSTD1* siRNA (si-*TSTD1*; Cat No.: 4390771, Ambion, USA) or 10 nM Silencer Select Negative Control No. 1 siRNA (Cat No. 4390843, Ambion) using Lipofectamine RNAiMAX transfection reagent (Cat No.: LMRNA015, Invitrogen, MA, USA). MDA-MB-231 and MCF7 cells were transfected with vector and pCMV-*TSTD1* using Lipofectamine 3000 (Invitrogen) according to the manufacturer’s protocol. Cells were incubated in original culture medium after transfection.

Different breast cancer cell lines were performed for different types of functional assays based on the expression level of *TSTD1* in each cell. Knockdown was performed in overexpression of TSTD1 in T47D. Conversely, transfection was performed at the low level of *TSTD1* in MDA-MB-231 and MCF7 cells.

### Plasmid extraction, confirmation, and purification

The extraction, confirmation, and purification of the TSTD1 Plasmid DNA was performed using the Geneaid™ Midi Plasmid Kit (Geneaid Biotech Ltd., Cat. No. PI025) according to the manufacturer’s instructions. The extracted DNA was subjected to preliminary length analysis by sequence to confirm errorless production. The plasmid concentration was measured using a NanoDrop 2000C ultramicrowavelength spectrophotometer (Thermo Fisher Scientific, USA), and the plasmid was stored at -20°C until further use.

### Cell proliferation assay

Twenty fours hours after treatment with either si-*TSTD1* or control siRNA, T47D and H1299 cells were counted using a microscope and hemocytometer. Experiments were performed in triplicate.

Twenty-four hours after pCMV-*TSTD1* plasmid transfection, MCF7 cells were treated with tamoxifen (10 and 20 μM; Cat No.: 13258, Cayman), epirubicin (100 and 2000 nM; Cat No.: 12091, Cayman), docetaxel (Cat No.: 11637, Cayman, MI, USA) or the vehicle control, dimethyl sulfoxide (DMSO; Cat No.: D2650, Sigma Aldrich), and incubated for another 24 h. Cell proliferation was determined by the MTT assay: 20 μL of MTT solution was added to each well; after 4 h, the MTT solution was removed and 100 μL DMSO was added. Optical density (OD) was measured by absorbance at 550nm by VarioskanFlash multimode reader (Thermo Fisher Scientific, Massachusetts, USA).

### Cell lysis and liquid chromatography–mass spectrometry analysis

Twenty-four hours after siRNA transfection, T47D cells were microcentrifuged and lysed at 4°C using the ultrasonic processor UP200H (Hielscher, Teltow, Germany) for 10 s. Proteins were precipitated from the cell lysis solution using acetonitrile. After centrifugation, the supernatant was immediately used for liquid chromatography–mass spectrometry (LC–MS) analysis.

For LC–MS, we Acquity UPLC binary pump coupled with a Xevo TQ-XS triple quadrupole mass spectrometer (Waters, MA, USA). An Acquity UPLC HSS T3 column was used to perform chromatographic separation (1.8 µm, 2.1 mm × 100 mm) at 40°C and a constant flow rate of 400 µL/min. The injection volume was 2 µL. The mobile phase consisted of 0.1% formic acid in water (A) and formic acid in 0.1% acetonitrile (B). The gradient program was set as follows: 0–3 min of 0%–100% of phase B and 3–4 min of isocratic elution with 100% of phase B. The column was re-equilibrated for 5 min by using 100% of phase A before analyzing the next sample. Target compounds and their respective derivatives were detected in both positive and negative modes. The capillary voltages were 3000 and 2000 V, respectively. The source and desolvation temperatures were set at 150°C and 450°C, respectively. During the assay, the mass spectrometer performed full scan cycles (*m/z* 200–1000). The data were compared with the reference of glutathione (GSH, molecular weight [MW]: 307.3) and glutathione disulfide (GSSG, MW 656.6). Experiments were conducted in triplicate.

### ROS detection

Subsequently, 24 h after siRNA transfection, T47D cells were treated with DCFH-DA and incubated for 30 min at 37°C. Pyocyanin was the positive control, whereas N-acetyl cysteine was the negative control. Fluorescence was measured at an excitation wavelength of 480 nm and an emission wavelength of 510 nm by the VarioskanFlash multimode reader (Thermo Fisher Scientific).

### Statistical analysis

All statistical analyses were performed using SPSS (SPSS Inc., Chicago, IL, USA). The relation between all the clinical data of the patients with breast cancer—namely age, sex, tumor type, TNM stage, metastasis condition, differentiation grade and location, recurrence status, and drug response and *TSTD1* molecular data (i.e. DNA methylation, mRNA expression and protein expression levels) was assessed using the Pearson’s chi-square test. The correlation between the DNA methylation and mRNA expression of *TSTD1* was measured using Pearson’s correlation. Differences in *TSTD1* mRNA expression between breast tumor tissues and adjacent normal tissues were confirmed by a paired student’s t-test. We also used the student’s t-test to compare cells transfected with or without *TSTD1* plasmid or si-*TSTD1* to controls and cells treated with drugs to controls.

## Results

### *TSTD1* was identified using genome-wide methylation analysis in Taiwanese patients with breast cancer and the Western TCGA cohort

**Figure 1:**
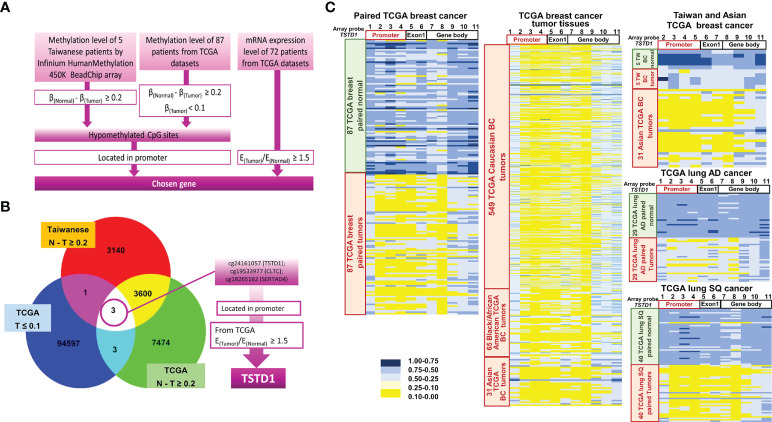
Flowchart of gene selection, analytical procedures and methylation heatmap for *TSTD1*. **(A)** The criteria and step-by-step flowchart for gene selection. **(B)** Screening of intersecting genes by InteractiVenn. **(C)** *TSTD1* methylation pattern in 87 paired TCGA breast cancer, 549 Caucasian, 65 Black/African and 31 Asian TCGA breast tumor tissues, *TSTD1* methylation level of 5 Taiwanese patients, TCGA paired lung adenocarcinoma and paired lung squamous-cell carcinoma by Methylation Beadchips array and EPIC Methylation Beadchips array.

The methylation arrays obtained from the Infinium HumanMethylation 450K and EPIC methylation array were used to determine the methylation status in five pairs of breast cancer tissues obtained from Taiwanese patients. In addition, the DNA methylation status of 87 tumor and normal tissue pairs of Western patients (Asian, Caucasian and Black/African Amercian) with breast cancer from TCGA datasets was collected. All the data were combined and analyzed using InteractiVenn by selecting the intersection list of aberrant hypomethylation CpG sites (**Figure 1A**). Three CpG sites of three genes were selected: cg24161057 located on the promoter of *TSTD1*, cg19533977 located in CLTC’s coding region, and cg18265162 located in on the promoter of SERTAD4. After excluding one gene whose CpG site is not located in the promoter, we examined the expression of the two other genes in 72 patients from TCGA. We determined that *TSTD1*, containing CpG site cg24161057 in the promoter, was overexpressed in mRNA sequencing data obtained from TCGA. Thus, *TSTD1* was selected for further examination (**Figure 1B**).

The methylation profiles for Taiwanese breast cancer and TCGA cohort of Asian and Western breast cancer population (Caucasian and Black/African) were analyzed. The analysis of data from TCGA and Taiwanese revealed significantly decreased *TSTD1* methylation levels in breast tumors compared with adjacent normal tissues. In addition, hypomethylation of *TSTD1* promoter was observed obviously in breast tumor tissues from Asian, Caucasian and Black/African American TCGA cohort compare with breast tumor tissues from Taiwanese cohort (**Figure 1C**), Further analysis of the methylation patterns of other cancer types indicated that *TSTD1* promoter hypomethylation was observed in lung adenocarcinoma and lung squamous cell carcinoma (**Figure 1C**) but not in liver, colon, esophageal, rectal, and pancreatic cancers (**Figure S2**).

### *TSTD1* promoter hypomethylation, mRNA and protein overexpression in breast cancer tissues obtained from Asian patients.

**Figure 2:**
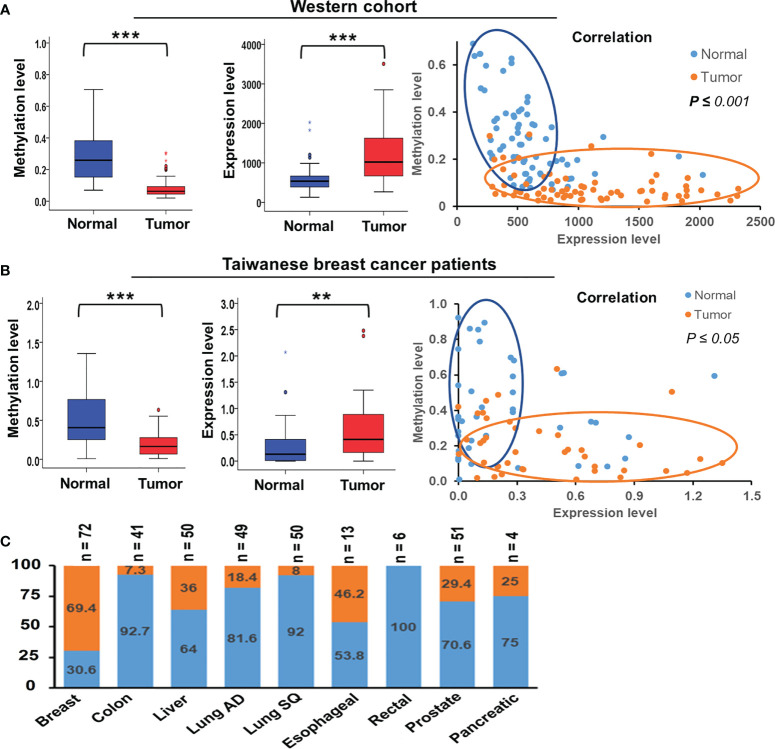
Methylation and expression of *TSTD1* in breast cancer and in various cancer types. **(A)** DNA methylation level, mRNA expression level and the correlation between *TSTD1* hypomethylation and overexpression in of tumor and adjacent normal tissues of the Western breast cancer cohort, analyzed from TCGA datasets. **(B)** DNA methylation level, mRNA expression level and the correlation between *TSTD1* hypomethylation and overexpression in of tumor and adjacent normal tissues of Taiwanese breast cancer patients. **(C)** mRNA expression level of *TSTD1* in various cancer types. The relationship was analyzed using Pearson X^2^ test. *ACTB* and *GAPDH* were the internal controls for DNA methylation and mRNA expression, respectively. Data were analyzed using student’s paired t-test. *p ≤ 0.05, **p ≤ 0.01, ***p ≤ 0.001.

We collected paired samples of cancerous and adjacent noncancerous tissue from Taiwanese patients with breast cancer for molecular studies to determine alterations in *TSTD1* methylation and expression. Hypomethylation of *TSTD1* was verified with qMSP in 106 samples (**Figure S3**). Of the 106 paired samples, methylation level in 69 tumor samples (65.09%) was less than 50% of that in the corresponding normal tissue (**Figure 2B**). *TSTD1* mRNA expression was measured using RT-qPCR (**Figure S4** and **Table 1**). Of the paired samples, 68.3% (43/63) exhibited higher *TSTD1* mRNA expression in tumor tissues than in the paired normal tissues (**Figure 2B**). In Taiwanese patients, methylation of *TSTD1* was significantly correlated with mRNA expression of *TSTD1*. *TSTD1* was hypomethylated in 74.2% (23/31) patients with upregulated *TSTD1* expression in breast tumors (*p =* 0.040), whereas *TSTD1* was hypermethylated in 56.3% (9/16) of patients with *TSTD1* downregulation (**Table S2**).

**Table 1:** TSTD1 protein expression, mRNA expression, and methylation levels in relation to the clinical parameters of Taiwanese patients with breast cancer^1^.

	Total n	Protein expression		Total n	mRNA expression^2^		Total n	DNA Methylation^3^	
		Low n (%)	High n (%)		Low n (%)	Highn (%)		Low n (%)	High n (%)
**Overall**	64	20 (31.3)	44 (68.8)	62	20 (32.3)	42 (67.7)	102	36 (35.3)	66 (64.7)
**Age**									
< 65	56	18 (32.1)	38 (67.9)	4	1 (25.0)	3 (75.0)	9	1 (11.1)	8 (88.9)^0.025^
≥ 65	8	2 (25.0)	6 (75.0)	5	2 (40.0)	3 (60.0)	6	4 (66.7)	2 (33.3)
**Type**									
ILC	1	0 (0.0)	1 (100.0)	0	0 (0.0)	0 (0.0)	2	1 (50.0)	1 (50.0)
IDC	58	18 (31.0)	40 (69.0)	62	20 (32.3)	42 (67.7)	102	35 (35.0)	65 (65.0)
**Stage**									
I, II	46	14 (30.4)	32 (69.6)	44	15 (34.1)	29 (65.9)	72	24 (33.3)	48 (66.7)
III, IV	16	5 (31.3)	11 (68.8)	16	5 (31.3)	11 (68.8)	27	8 (29.6)	19 (70.4)
**T**									
T0-T1	13	3 (23.1)	10 (76.9)	17	6 (35.3)	11 (64.7)	30	15 (50.0)	15 (50.0)
T2-T4	48	16 (33.3)	32 (66.7)	43	14 (32.6)	29 (67.4)	70	18 (25.7)	52 (74.3)^0.018^
**N**									
N0	27	6 (22.2)	21 (77.8)	27	8 (29.6)	19 (70.4)	45	14 (31.1)	31 (68.9)
N1-N3	8	3 (37.5)	5 (62.5)	32	11 (34.4)	21 (65.6)	51	18 (35.3)	33 (64.7)
**M**									
M0	63	20 (31.7)	43 (68.3)	34	13 (38.2)	21 (61.8)	73	20 (27.4)	53 (72.6)
M1	1	0 (0.0)	1 (100.0)	0	0 (0.0)	0 (0.0)	0	0 (0.0)	0 (0.0)
**ER**									
Negative	29	15 (51.7)	14 (48.3)	18	4 (22.2)	14 (77.8)	33	14 (42.4)	19 (57.6)
Positive	24	0 (0.0)	24 (100.0)^<0.001^	34	12 (35.3)	22 (64.7)	67	20 (29.9)	47 (70.1)
**PR**									
Negative	32	12 (37.5)	20 (62.5)	20	5 (25.0)	15 (75.0)	44	18 (40.9)	26 (59.1)
Positive	21	3 (14.3)	18 (85.7)	32	11 (34.4)	21 (65.6)	56	16 (28.6)	40 (71.4)
**HER2**									
Negative	37	11 (29.7)	26 (70.3)	26	8 (30.8)	18 (69.2)	42	16 (38.1)	26 (61.9)
Positive	16	4 (25.0)	12 (75.0)	27	7 (28.0)	18 (72.0)	57	18 (31.6)	39 (68.4)
**TNBC**									
No	37	6 (16.2)	31 (83.8)	39	11 (28.2)	28 (71.8)	81	26 (32.1)	55 (67.9)
Yes	16	9 (56.3)	7 (43.8)	12	4 (33.3)	8 (66.7)	18	8 (44.4)	10 (55.6)
**Ki-67**									
Negative	2	0 (0.0)	2 (100.0)	12	3 (25.0)	9 (75.0)	23	12 (52.2)	11 (47.8)
Positive	11	1 (9.1)	10 (90.9)	31	12 (38.7)	19 (61.3)	70	18 (25.7)	52 (74.3)^0.019^
Luminal A									
No	6	2 (33.3)	4 (66.7)	35	10 (28.6)	25 (71.4)	72	45 (62.5)	27 (37.5)
Yes	7	2 (28.6)	5 (71.4)	14	4 (28.6)	10 (71.4)	23	19 (82.6)	4 (17.4)
Luminal B									
No	9	3 (33.3)	6 (66.7)	28	8 (28.6)	20 (71.4)	52	37 (71.2)	15 (28.8)
Yes	4	1 (25)	3 (75)	21	6 (28.6)	15 (71.4)	43	27 (62.8)	16 (37.2)

*, p < 0.05; **, p < 0.01; ***, p < 0.001.

^1^These results were analyzed by the Pearson’s X^2^ test. P values with significance are shown as superscripts.

^2^TSTD1 was considered high when the TSTD1 expression level was more than one and a half in breast tumors compared to adjacent normal breast tissues.

^3^TSTD1 methylation was considered hypomethylation when the TSTD1 methylation level was less than half in breast tumors compared to adjacent normal breast tissues.

**Figure 3:**
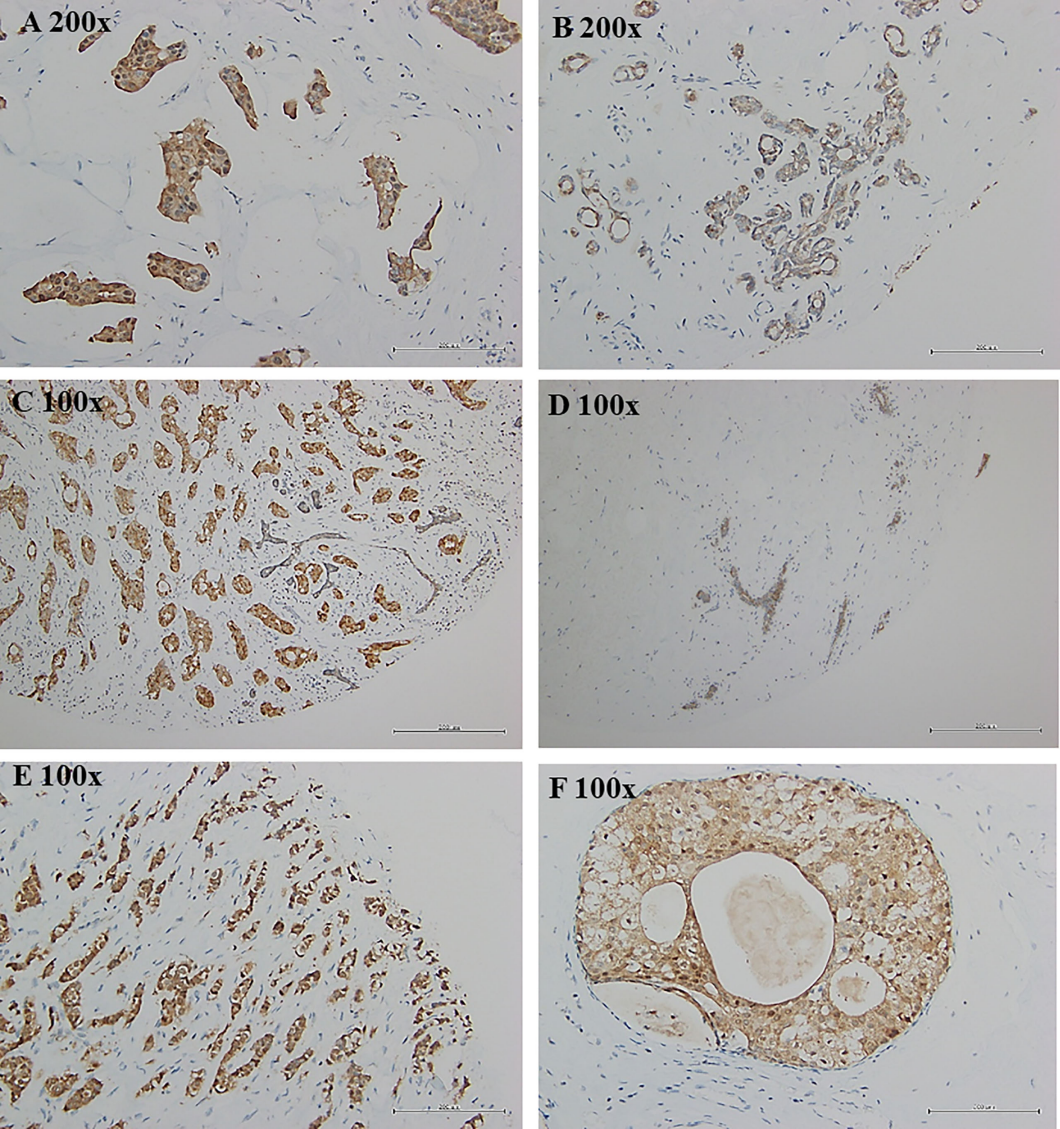
Protein expression of tstd1 in breast tissues. **(A)** tumor tissues; **(B, D)** normal/lobular tissues; **(C, E)** invasive tumor tissues; **(F)** carcinoma *in situ* tissues. Immunohistochemistry staining results were captured by microscope. Images in the same row were taken from same patient. **(A, B)** patient sh078; **(C, D)** patient sh085; **(E, F)** patient sh095. Original magnification is as shown in each image.

Breast tumor samples from Taiwanese and Korean breast cancer patients were stained with a TSTD1 antibody for immunohistochemical analysis. High intensity of TSTD1 protein staining was observed in 68.8% (44/64) of the patients (**Table 1**). Within cancerous tissues, TSTD1 staining was more robust in invasive areas than ductal carcinoma in situ. In addition, there was moderate staining of TSTD1 near lobules and duct (**Figure 3**). In general, the intensity of TSTD1 staining increased from normal to lobular to carcinoma *in situ* tissues, becoming the highest in invasive carcinoma tissues.

By combining protein expression levels with clinical information data, we determined that all patients with positive estrogen receptor (ER) exhibited high protein expression (24/24, *p ≤* 0.001). A larger sample size is required to make a definite conclusion.

### *TSTD1* promoter hypomethylation and mRNA overexpression in breast cancer tissues from the TCGA dataset

To determine alteration in *TSTD1* hypomethylation and mRNA expression in Western patients with breast cancer, we analyzed the TCGA data of 87 breast tumors, 87 matched normal tissues, and 643 breast tumor tissues. First, we analyzed the Illumina Infinium HumanMethylation450 BeadChip array data (**Figure S2**). The CpG site cg24161057, which is on the promoter of *TSTD1*, was hypomethylated in 77.0% (67/87) of breast paired tissues and 81.0% (521/643) of non-paired breast tumor tissues. Analysis of RNA sequencing data obtained from TCGA revealed that 69.4% (50/72) of patients with breast cancer had significant *TSTD1* mRNA upregulation in tumors compared with adjacent normal tissues.

Overexpression of *TSTD1* mRNA was associated with the histological type and tumor stage of patients with breast cancer (*p* < 0.001 and 0.040, respectively, **Table 2**). Overexpression of *TSTD1* mRNA was associated with negative ER and negative progesterone receptor (PR) expression in patients with breast cancer (*p < 0.001*, **Table 2**). A multivariate Cox proportional hazards survival analysis revealed that *TSTD1* mRNA expression in patients with breast cancer was significantly associated with poor 5-year overall survival in TCGA data (*p* = 0.021, **Table 3**), especially in patients with negative ER/PR expression (*p* = 0.008, **Table 3**). TSTD1 methylation was significantly associated with mRNA expression of *TSTD1* in Western patients. We found that 44 (88%, *p <* 0.001) of 50 patients with higher *TSTD1* expression demonstrated hypomethylation (**Figure 2A**; **Table S2**).

**Table 2:** *TSTD1* mRNA expression and methylation levels in relation to the clinical parameters of Western with breast cancer.

Characteristics	Total n	*TSTD1* mRNA^2^		Total n	*TSTD1* Methylation^3^	
		Low n (%)	High n (%)		Low n (%)	High n (%)
Overall	408	142 (34.8)	266 (65.2)	624	573 (91.8)	51 (8.2)
Age (years)						
<65	295	104 (35.3)	191 (64.7)	453	418 (92.3)	35 (7.7)
≥65	113	38 (33.6)	75 (66.4)	170	155 (91.2)	15 (8.8)
Race						
White	312	111 (35.6)	201 (64.4)	338	308 (91.1)	30 (8.9)
Black/African American	72	19 (26.4)	53 (73.6)	65	61 (93.8)	4 (6.2)
Asian	6	3 (50.0)	3 (50.0)	31	27 (87.1)	4 (12.9)
Menopause State						
Premenopause	97	38 (39.2)	59 (60.8)	103	99 (96.1)	4 (3.9)
Perimenopause	10	5 (50.0)	5 (50.0)	14	9 (64.3)	5 (35.7)
Postmenopause	233	78 (33.5)	155 (66.5)	287	258 (89.9)	29 (10.1)
Histological Type						
ILC	98	18 (18.4)	80 (81.6)^<0.001^	152	131 (86.2)	21 (13.8)
IDC	262	102 (38.9)	160 (61.1)	400	379 (94.8)	21 (5.3)
Mixed type	29	14 (48.3)	15 (51.7)	41	34 (82.9)	7 (17.1)
Tumor Stage						
I and II	279	108 (38.7)	171 (61.3)^0.040^	311	257 (82.6)	54 (17.4)
III and IV	115	32 (27.8)	83 (72.2)	129	100 (77.5)	29 (22.5)
Tumor size						
T0–T1	103	37 (35.9)	66 (64.1)	295	268 (90.8)	27 (9.2)
T2–T4	305	105 (34.4)	200 (65.6)	329	305 (92.7)	24 (7.3)
Lymph nodes						
N0	183	71 (38.8)	112 (61.2)	382	350 (91.6)	32 (8.4)
N1–N3	225	71 (31.6)	154 (68.4)	242	223 (92.1)	19 (7.9)
Metastasis						
M0	399	136 (34.1)	263 (65.9)	617	566 (91.7)	51 (8.3)
M1	9	6 (66.7)	3 (33.3)	7	7 (100.0)	0 (0.0)
ER						
Negative	89	50 (56.2)	39 (43.8)^<0.001^	103	94 (91.3)	9 (8.7)
Positive	307	88 (28.7)	219 (71.3)	340	311 (91.5)	29 (8.5)
PR						
Negative	133	64 (48.1)	69 (51.9)^<0.001^	143	130 (90.9)	13 (9.1)
Positive	262	73 (27.9)	189 (72.1)	299	274 (91.6)	25 (8.4)
HER2						
Negative	214	75 (35.0)	139 (35.0)	247	221 (89.5)	26 (10.5)
Positive	43	15 (34.9)	28 (65.1)	45	43 (95.6)	2 (4.4)
TNBC						
No	210	62 (29.5)	148 (70.5)	238	218 (91.6)	20 (8.4)
Yes	46	27 (58.7)	19 (41.3)	53	45 (84.9)	8 (15.1)
Luminal A						
No	89	42 (47.2)	47 (52.8))^0.004^	98	88 (89.8)	10 (10.2)
Yes	167	47 (28.1)	120 (71.9)	193	175 (90.7)	18 (9.3)
Luminal B						
No	204	72 (35.3)	132 (64.7)	230	207 (90)	23 (10)
Yes	52	17 (32.7)	35 (67.3)	61	56 (91.8)	5 (8.2)

*p < 0.05; **p < 0.01; ***p < 0.001.

^1^These results were analyzed by the Pearson X^2^ test. P values with significance are shown as superscripts.

^2^TSTD1 was considered high when the TSTD1 expression level was more than one and a half in breast tumors compared to adjacent normal breast tissues.

^3^TSTD1 methylation was considered hypomethylation when the TSTD1 methylation level was less than half in breast tumors compared to adjacent normal breast tissues.

Data generated from TCGA datasets^1^.

**Table 3:** Cox proportional hazard model of clinical parameters and TSTD1 mRNA expression level in TCGA breast cancer.

Variable	Multivariate analysis of 5-year overall survival^1^					
	All BC patients			Negative ER/PR		
	HR	95% CI	*P*-value	HR	95% CI	*P*-value
Race	1.217	0.376-3.937	0.743	0.716	0.715-2.934	0.643
Age	1.053	1.005-1.105	0.032	1.044	0.988-1.103	0.129
Tumor type	0.996	0.406-2.441	0.992	0.971	0.332-2.839	0.957
Tumor size	0.606	0.290-1.268	0.184	0.621	0.281-1.371	0.238
lymph nodes	1.255	0.767-1.957	0.395	1.178	0.687-.2.020	0.551
Stage	2.797	1.600-4.890	<0.001	3.228	1.682-6.194	<0.001
Menopause	1.332	0.521-3.405	0.549	1.218	0.456-3.294	0.694
TSTD1 mRNA expression^3^	1.001	1.000-1.001	0.021	1.001	1.000-1.001	0.008

*p < 0.05; **p < 0.01; ***p < 0.001.

^1^These results were analyzed by the Cox regression model.

^2^BC = breast cancer; Negative ER/PR = breast cancer patients with negative expression of estrogen receptor (ER) and progesterone receptor (PR).

^3^The TSTD1 mRNA expression levels were derived from RNA sequencing data of 754 breast cancer patients in TCGA data set.

### *TSTD1* expression was involved in cell proliferation in breast cancer and lung cancer cell lines

**Figure 4:**
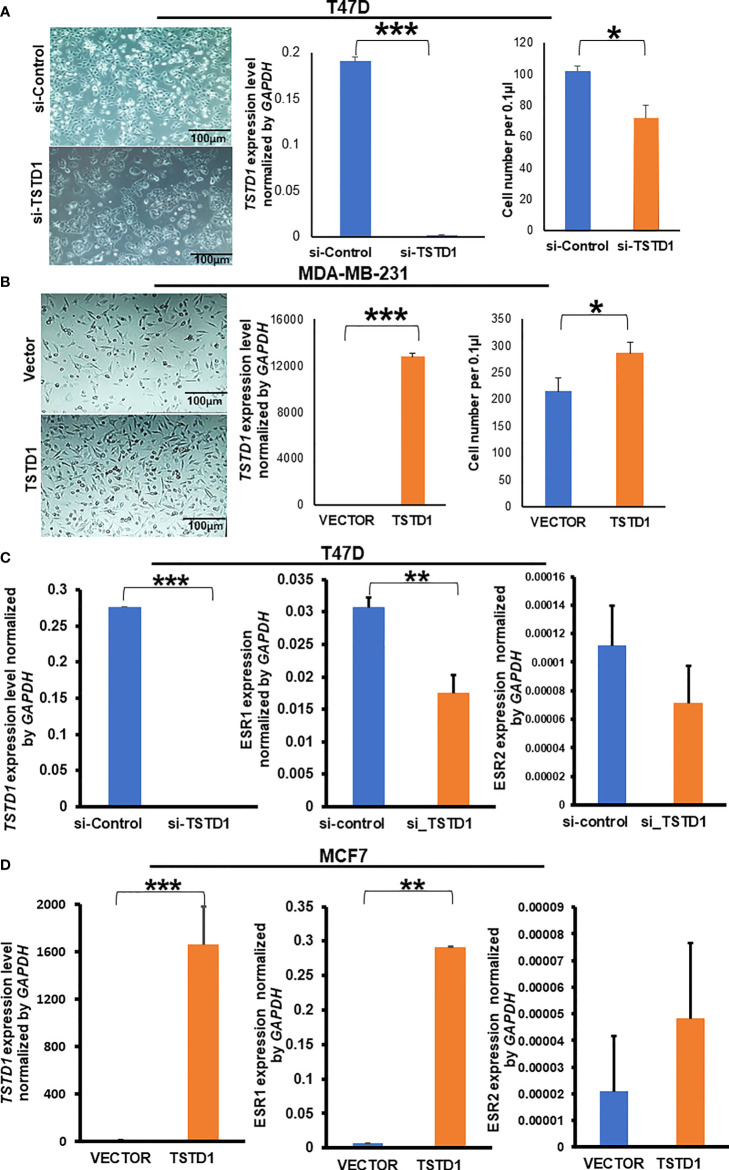
*TSTD1* expression repressed and induced the proliferation of cancer cell lines and involved in the alteration of *ESR1* and *ESR2*. GAPDH was used as internal control, **(A)** Images of T47D breast cancer cell lines after transfection with si-TSTD1 and the relative proliferation of T47D cells, **(B)** expression of *TSTD1* and the relative proliferation of MDA-MB-231 cells after pCMV-*TSTD1* plasmid transfection, **(C)** *ESR1* and *ESR2* expression after knockdown of *TSTD1* in T47D cell line, **(D)** *ESR1* and *ESR2* expression after pCMV-*TSTD1* plasmid transfection in MCF7 cell line. The data are presented as the mean ± SD, *p ≤ 0.05, **p ≤ 0.01, ***p ≤ 0.001.

Hypomethylation of *TSTD1* was observed in Western and Taiwanese breast and lung cancer patients. However, overexpression of *TSTD1* mRNA expression only was found in breast cancer patients (**Figure 2C**), but not in lung cancer patients. To further analyze whether the *TSTD1* expression level was involved in breast and lung cancer growth, *TSTD1* gene manipulation was conducted in breast and lung cancer cell lines. RT-qPCR was conducted to evaluate the expression of *TSTD1* in breast and lung cell lines. *TSTD1* expression was significantly higher by 47-fold in T47D cells (*p* < 0.001, **Figure 4A**), and 3-fold in H1299 cells compared to normal breast and lung cells (*p* < 0.001, **Figure S5**). Therefore, T47D and H1299 cell lines were used for knockdown experiments. T47D cells treated with si-*TSTD1* exhibited a 30% lower proliferation rate than the control group (*p=0.04*, **Figure 4A**) after 24 hours. By contrast, although H1299 cells appeared to grow rapidly, they demonstrated a 28% decrease in cell number after *TSTD1* knockdown than the control group (*p= 0.04*, **Figure S5**).

After transfection of the pCMV-*TSTD1* plasmid, MDA-MB-231 cells abundantly expressed *TSTD1* mRNA (*p*< 0.001, **Figure 4B**). Cell counting and microscopic observation revealed that *TSTD1* overexpression induced increase by 27% of the growth of MDA-MB-231 cells compared with a vector control (*p*=0.012, **Figure 4B**).

### *TSTD1* expression involved in ESR1 and ESR2 levels

RT-qPCR revealed the expression levels of Estrogen Receptor 1 (*ESR1)* decreased by 0.5-fold in T47D cells after *TSTD1* knockdown (*p*=0.002, **Figure 4C**). The expression levels of *ESR1* increased by 43-fold in MCF7 cells after *TSTD1* transfection (*p*=0.003, **Figure 4D**). The results are consistent with the clinical data, where *TSTD1* overexpression in Western and high *TSTD1* protein expression in Taiwanese are correlate with increased estrogen levels in patients with breast cancer (**Tables 1**, **2**).

### *TSTD1* expression involved in chemotherapy and hormone therapy drug responses

To determine whether *TSTD1* expression is involved in responses to clinical treatment, we analyzed clinical data from TCGA datasets. High *TSTD1* mRNA expression was correlated with the poor drugs treatment response in Western patients with breast cancer patients (*p =* 0.030, **Table 4**), especially for chemotherapy drug response (*p =* 0.005). Patients with complete responses were more likely to have tumors with *TSTD1* downregulation before treatment (69 of 111 patients), whereas those who experienced progressive disease were more likely to have tumors with *TSTD1* upregulation before treatment (9 of 11 patients).

**Table 4:** *TSTD1* mRNA expression and methylation level in relation to the drug response of Western breast cancer patients in TCGA datasets ^1^.

Characteristic	Total n	Expression						Methylation			
			Low n (%)	High n (%)		Total n		Low n (%)		High n (%)	
**Overall**	141		83 (58.9)	58 (41.1)^0.030^		272		202 (74.3)		70 (25.7)	
PD	14	3 (21.4)		11 (78.6)		25		19 (76.0)		6 (24.0)	
CR	127	80 (63.0)		47 (37.0)		247		183 (73.1)		64 (26.9)	
**Chemotherapy^2^**										
PD	11	2 (18.2)		9 (81.8)^0.005^		14		11 (78.6)		3 (21.4)	
CR	111	69 (62.2)		42 (37.8)		216		164 (75.9)		52 (24.1)	
** Alkylating drugs**											
PD	2	0 (0.0)		2 (100.0)		2		1 (50.0)		1 (50.0)	
CR	43	26 (60.5)		17 (39.5)		77		58 (75.3)		19 (24.7)	
** Antimetabolites**											
PD	3	0 (0.0)		3 (100.0)^0.014^		5		5 (100.0)		0 (0.0)	
CR	3	3 (100.0)		0 (0.0)		25		19 (76.0)		6 (24.0)	
** Topoisomerase inhibitors**											
PD	1	0 (0.0)		1 (100.0)		1		0 (0.0)		1 (100.0)	
CR	28	17 (60.7)		11 (39.3)		51		39 (76.5)		12 (23.5)	
** Microtubule Inhibitors**											
PD	5	2 (40.0)		3 (60.0)		4		4 (100.0)		0 (0.0)	
CR	20	13 (65.0)		7 (35.0)		35		25 (71.4)		10 (28.6)	
** Microtubule Stabilizers**											
PD	0	0 (0.0)		0 (0.0)		2		1 (50.0)		1 (50.0)	
CR	17	10 (58.8)		7 (41.2)		28		23 (82.1)		5 (17.9)	
**Hormone Therapy^3^**										
PD	3	1 (33.3)		2 (66.7)		11		8 (72.7)		3 (27.3)	
CR	15	10 (66.7)		5 (33.3)		30		19 (63.3)		11 (36.7)	
** Estrogen Inhibitors**											
PD	1	0 (0.0)		1 (100.0)		6		4 (66.7)		2 (33.3)	
CR	2	1 (50.0)		1 (50.0)		8		6 (75.0)		2 (25.0)	
** Aromatase inhibitors**											
PD	2	1 (50.0)		1 (50.0)		5		4 (80.0)		1 (20.0)	
CR	5	4 (80.0)		1 (20.0)		13		8 (61.5)		5 (38.5)	
** Immunotherapy**										
PD	0	0 (0.0)		0(0.0)		0		0 (0.0)		0 (0.0)	
CR	1	1 (100.0)		0 (0.0)		1		0 (0.0)		1 (100.0)	
**Target Therapy**											
** HER2 inhibitors**											
PD	0	0 (0.0)		0 (0.0)		0		0 (0.0)		0 (0.0)	
CR	8	5 (62.5)		3 (37.5)		9		5 (55.6)		4 (44.4)	

*p < 0.05; **p < 0.01; ***p < 0.001.

^1^These results were analyzed by the Pearson X^2^ test. P values with significance are shown as superscripts. The patients with a treatment duration of greater than 4 weeks were included in this analysis. After using RNA sequencing analysis, TSTD1 was considered high when the TSTD1 expression level was more than one and a half in breast tumors compared to adjacent normal breast tissues. After using Illumina 450K, TSTD1 methylation was considered hypomethylation when the TSTD1 methylation level was less than half in breast tumors compared to adjacent normal breast tissues.

^2^PD, Clinical Progressive Disease; CR, Complete Response.

^3^Antimetabolites drugs: 5-fluorouracil, capecitabine, gemcitabine, methotrexate; alkylating drugs: cyclophosphamide, cisplatin, carboplatin and trabectidin; topoisomerase inhibitors: doxorubicin, mitoxantrone and epirubicin; microtubule inhibitors: docetaxel and vinca alkaloids; microtubule stabilizer: paclitaxel and ixabepilone.

^4^Estrogen inhibitors: tamoxifen, goserelin, leuprolide and fulvestrant; aromatase inhibitors: letrozole, anastrozole and exemestane; HER2 tyrosine kinase inhibitors: trastuzumab.

**Figure 5:**
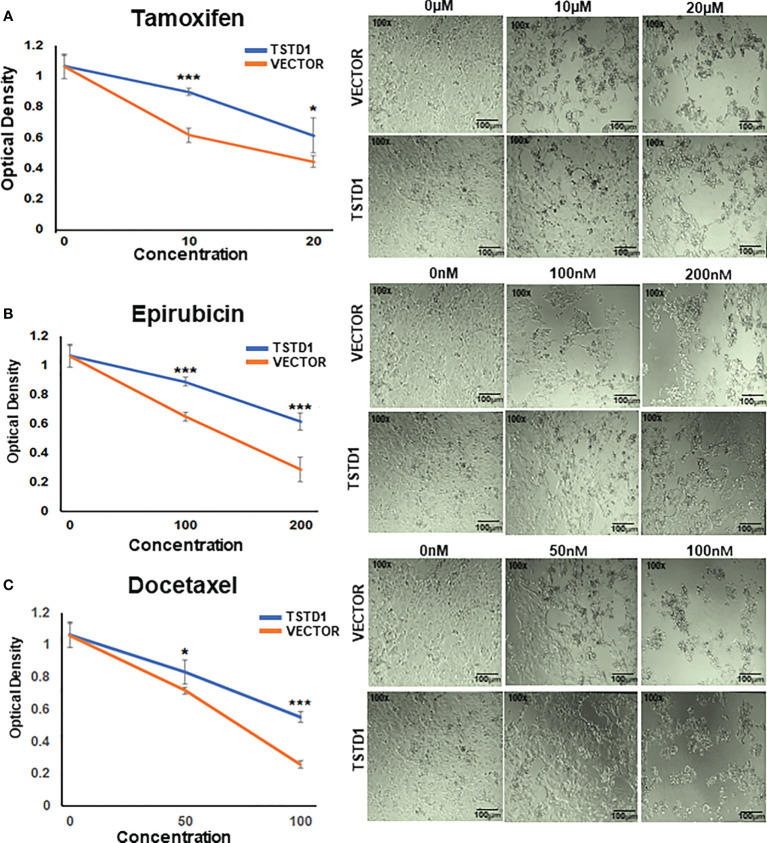
*TSTD1* overexpression mediated poor treatment response. Cell proliferation assays were performed in MCF7 cells treated with **(A)** Tamoxifen, **(B)** Epirubicin, and **(C)** Docetaxel after TSTD1 plasmid transfection, in a dose-dependent manner. The result was analyzed using students paired t-test. The data are presented as the means ± SD. *p ≤ 0.05, **p ≤ 0.01, ***p ≤ 0.001.

*TSTD1* overexpression in MCF7 cells consistently and significantly mediated a poor response to chemotherapy and hormone therapy in a dose-dependent manner (**Figure 5**). Overexpression of *TSTD1* in MCF7 decreased epirubicin-mediated toxicity. When treated with 200 nM of epirubicin, 42.5% of cells transfected with TSTD1 were decreased compared the 72.9% of cells in the vector control (*p*< 0.001). **O**verexpression of *TSTD1* in MCF7 also decreased the toxicity of 100 nM of docetaxel: 48% of the cells were reduced compared to the 75.5% of cells in the vector control (*p*< 0.001). Furthermore, when treated with 20 mM of tamoxifen, 42.4% of cells were decreased in conditions of overexpressed *TSTD1*, compared the 58.1% of cells in the vector control (*p*= 0.025, **Figure 5A**). The results are consistent with the TCGA cohort data, where *TSTD1* overexpression is correlated with poor drugs treatment response in breast cancer patients (**Table 4**).

### Knockdown of *TSTD1* increased GSH/GSSG and reduced ROS

A previous study reported that recombinant *TSTD1* catalyzes the reaction between glutathione (GS^−^) and thiosulfate (S2O32^−^) that forms sulfide (SO32^−^) and glutathione persulfide (GSS^−^) in the sulfide oxidation pathway (14). Another study suggested TSTD1 has a role in sulfide signaling (13). In this study, liquid chromatography–mass spectrometry LC–MS was used to determine whether *TSTD1* is involved in glutathione-dependent sulfide oxidation reaction in the T47D breast cancer cell line. The concentration of GSH and Glutathione disulfide (GSSG) in cell lysates was measured through LC–MS after treatment with si-*TSTD1* or siRNA controls. There was a 2.95-fold increase in the GSH–GSSG ratio detected in cells with *TSTD1* knockdowned through siRNA (*p*=0.003, **Figure S6**). Interestingly, knockdown of *TSTD1* also reduced ROS levels by 0.8-fold (*p*=0.016, **Figure S6B**).

### Circulating cell-free hypomethylated *TSTD1* was detected in Taiwanese breast cancer patients with disease progression and poor chemotherapy efficacy

**Figure 6:**
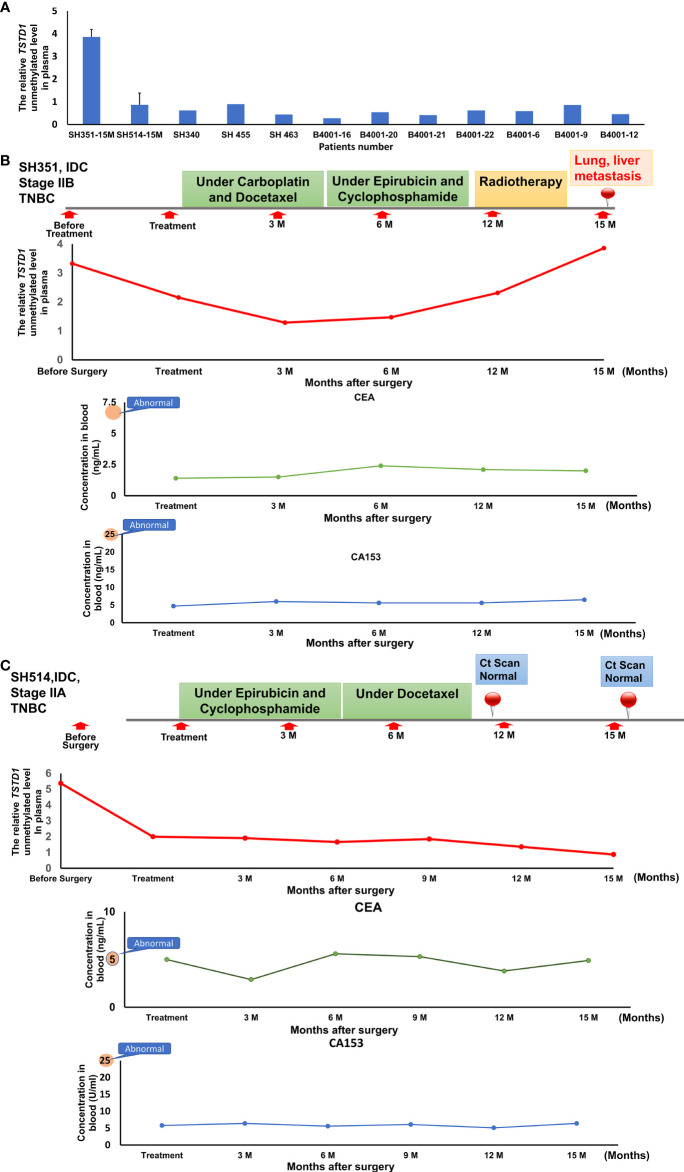
The circulating hypomethylated *TSTD1* was monitored in breast cancer patients. **(A)** The circulating cell-free hypomethylated TSTD1 can be detected in plasma of Taiwanese breast cancer patients. **(B)** Distance metastasis was found in triple-negative breast cancer patients after circulating hypomethylated *TSTD1* was gradually increased and revealed poor chemotherapy efficacy. The concentrations of CEA and CA15-3 in serum remained normal. **(C)** The lower level of circulating hypomethylated *TSTD1* was found in triple-negative breast cancer patients with better proficient treatment response and good prognosis.

To analyze whether hypomethylation *of TSDT1* can be detected in plasma and response to treatment, the hypomethylation (unmethylation) circulating cell-free *TSTD1* was measured. After these patients received treatment, circulating DNA was extracted from their plasma at 3-6 month intervals and analyzed by QMSP. Circulating cell-free hypomethylated *TSTD1* can be detected in all the breast cancer patients (n=12, **Figure 6A**). Circulating cell-free hypomethylated *TSTD1* was found to be higher in breast tumors of patients who displayed distant metastasis after treatment (**Figure 6B**). However, it was revealed that the level of circulating hypomethylated *TSTD1* was lower in patients with good prognosis after treatment (**Figure 6C**). The concentrations of CEA and CA15-3 in all of the patients serum remained normal and can therefore not be used to monitor disease progression and treatment response.

## Discussion

Previous studies have shown *TSTD1* is highly expressed in breast cancer cell lines but not in normal breast cell lines (32). No clinical study has reported whether alterations of this gene correlate with the progression and treatment response of breast cancer patients. Here we found the protein expression level of TSTD1 is significantly higher in invasive breast tumors of Taiwanese and Korean breast cancer patients. This study examined the methylation and gene product expression of *TSTD1* in Taiwanese and Western patients with breast cancer. We found overexpression of *TSTD1* mRNA is correlated with DNA hypomethylation in Taiwanese and Western patients. Furthermore, through analyzing TCGA datasets, we found promoter hypomethylation and overexpression of *TSTD1* in patients with breast cancer, even in Asian patients with breast cancer. Notably, hypomethylation of *TSTD1* was stronger in TCGA Asian patients with breast cancer compared with Taiwanese patients with breast cancer, most likely due to technological differences or environmental interference in the different countries. These findings suggest the alterations of *TSTD1* are potential tumor-specific biomarkers for breast cancer.

Upon examining the alterations of *TSTD1* in different types of cancer, we found *TSTD1* was hypomethylated in lung cancer samples from both the TCGA cohort and Taiwanese patients (**Figures 1C**, **S7**). However, no difference in *TSTD1* expression was observed between lung cancer tumor tissues and normal tissues. Therefore, we concluded that the relationship between *TSTD1* expression and methylation in patients with lung cancer depended on other conditional factors in the individual. Additionally, a study reported *TSTD1* expression in lung cancer cell models (32). In our study, siRNA knockdown of *TSTD1* in the H1299 lung cancer cell line suppressed cell growth (**Figure S5**). Additional clinical studies with a larger sample size are needed to provide a detailed analysis of the function of *TSTD1* in lung cancer.

Immunohistochemical analysis revealed strong expression of TSTD1 protein in the tumor tissue of patients with breast cancer; however, weak to no staining was noted in normal tissues (**Figure 3**). Additionally, Taiwanese patients with ER-positive cancer were observed a high expression level of TSDT1 protein (**Table 1**). In Western cohorts, most of the patients with ER- and PR-positive cancer exhibited *TSTD1* overexpression (**Table 2**). In addition, *ESR1* expression significantly decreased after *TSTD1* knockdown and significantly increased after transfection of *TSTD1* in separate breast cancer cell lines. Interestingly, more than half of all breast cancers that overexpress ER-alpha are associated with cellular proliferation (43, 44). Breast cancer cells often dependent on estrogen to grow and proliferate (45). These results together suggest that TSTD1 involved in the proliferation of breast cancer cells is partly to be mediated by positive regulation of ESR1 expression. In addition, a previous study revealed that *TSTD1* was the only hypomethylated gene related to HER2+ breast cancer patients (46). No further studies were undertaken. Furthermore, our clinical data in both Taiwanese and Western patients showed that there was no correlation between a *TSTD1* methylation profile in HER2+ breast cancer patients.

Glutathione (GSH), which plays a vital role in cell biology, is involved in drug metabolite detoxification, apoptosis regulation, cancer progression, drug sensitivity, and chemoresistance (47, 48). GSH levels are higher in breast tumor tissues than in adjacent cancer-free tissues (49, 50). Inhibition of GSH led to the death of cancer cells *in vitro* and *in vivo* (51). Our LC–MS revealed an increase in the GSH–GSSG ratio after *TSTD1* knockdown (**Figure S6A**). This result is supported by a study emphasizing the role of *TSTD1* in the glutathione-dependent sulfide oxidation pathway (31). *TSTD1* forms thiosulfate from glutathione persulfide (30). This is most likely why decreased ROS levels were observed after *TSTD1* knockdown in breast cancer cells (**Figure S6B**). In the presence of estrogen and moderate ROS levels, estrogen can generate ROS, thus exerting pro-proliferative and anti-apoptotic effects on breast cancer cells (52). ROS induced by estrogens initiate carcinogenesis and promote cancer transformation (53). These previous studies support the idea that decreases in estrogen and ROS levels are the mechanism for decreased breast cancer cell proliferation after TSTD1 knockdown.

Tamoxifen and aromatase inhibitors are hormonal therapies used in the treatment of ER- or PR-positive breast cancers to cease tumor growth and recurrence. Adjuvant endocrine therapy can reduce the risk of recurrence or improve breast cancer outcomes (54, 55). According to laboratory and clinical investigations, some chemotherapeutic and hormone therapy agents are less effective in ER-positive tumors than ER-negative tumors since estrogen reverses the effects of chemotherapy and hormone therapy (56–58). This study has shown that *TSTD1* expression decreases the cytotoxicity of epirubicin and docetaxel, which is consistent with clinical data from TCGA; patients with high expression *TSTD1* displayed poorer 5-year survival than those with low expression of *TSTD1*. We also found the poor response to tamoxifen in cells overexpressing TSTD1. In addition, hypomethylation of *TSTD1* in cell-free DNA can be detected in breast cancer patients with poor chemotherapy efficacy and disease progression. The mechanism of the poor drug response and resistance induced by TSTD1 is worthy of further investigation. In conclusion, TSTD1 is a potential biomarker for cancer outcomes: alterations of *TSTD1* could indicate poor drug treatment response and 5-year survival.

## Conclusions

**Figure 7:**
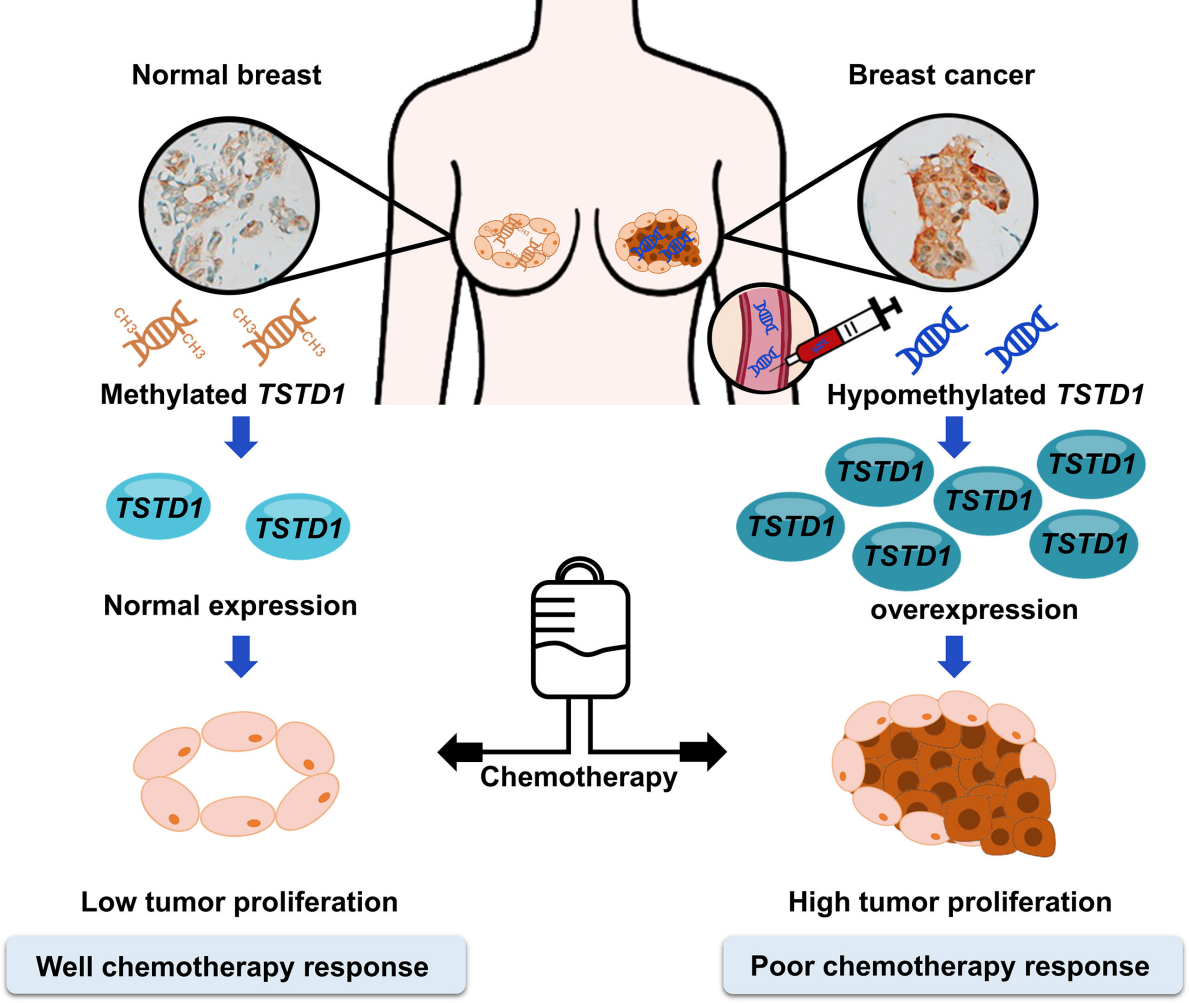
Promoter hypomethylation and overexpression of TSTD1 mediate poor treatment response in breast cancer.

Hypomethylation and overexpression of *TSTD1* were found specifically in the breast cancer tissues of patients in Asain and Western countries. Upregulation of *TSTD1* gene was mediated by DNA hypomethylation on the promoter of *TSTD1*. In addition, overexpression of TSTD1 was involved in breast cancer cell proliferation and poor chemotherapy and poor hormone therapy responses in breast cancer cells and breast cancer patients (**Figures 7**). Finally, the hypomethylation and overexpression of *TSTD1* are potential biomarkers for tumor proliferation and chemotherapy and hormone therapy drug response.

## Data availability statement

The data generated in this study are available from the corresponding author upon reasonable request.

## Ethics statement

The studies involving human participants were reviewed and approved by Taipei Medical University - Joint Institutional Review Board and the Institutional Review Board. The patients/participants provided their written informed consent to participate in this study. Written informed consent was obtained from the individual(s) for the publication of any potentially identifiable images or data included in this article.

## Author contributions

R-KL: Conceptualization. MA, LT, and Y-MC: Methodology. LT: Formal Analysis. LT, MA, and M-HH: Investigation. C-SH, C-MS, and L-ML: Resources. MA: Data Curation. LT, and MA: Writing – Original Draft Preparation. R-KL and LT. Writing – Review and Editing R-KL and C-SH: Supervision, R-KL, C-SH, and C-MS: Project Administration. R-KL: Funding Acquisition. All authors have read and agreed to the published version of the manuscript.

## Funding

This work was supported in part by the Ministry of Science and Technology, Republic of China, grant number MOST110-2622-B-038-007 and MOST108-2320-B-038-020 and Ministry of Education, Republic of China, grant number 110-5804-001-400.

## Acknowledgments

The authors are grateful for the support of the Core Facility Center of Taipei Medical University.

## Conflict of interest

The authors declare that the research was conducted in the absence of any commercial or financial relationships that could be construed as a potential conflict of interest.

## Publisher’s note

All claims expressed in this article are solely those of the authors and do not necessarily represent those of their affiliated organizations, or those of the publisher, the editors and the reviewers. Any product that may be evaluated in this article, or claim that may be made by its manufacturer, is not guaranteed or endorsed by the publisher.

## Abbreviations

ACTB, Beta-actin; GAPDH, Glyceraldehyde 3phosphate dehydrogenase; QMSP, Quantitative methylation-specific polymerase chain reaction; RT-qPCR, Reversetranscription quantitative polymerase chain reaction; TSTD1, Thiosulfate sulfurtransferate-like domain containing 1; BC, Breast cancer; TCGA, The Cancer Genome Atlas; GSH, Glutathione; GSSG, Glutathione disulfide; ESR1, Estrogen receptor 1; and ESR2, Estrogen receptor 2; ROS, Reactive oxygen species; TMU, Taipei Medical University.
