# Supplementary material for: Promoter hypomethylation and overexpression of TSTD1 mediate poor treatment response in breast cancer
Source: Front Oncol. 2022 Nov 7;12:1004261. doi: 10.3389/fonc.2022.1004261 (PMC9676938; doi:10.3389/fonc.2022.1004261)
Supplement: Supplementary file 1 [file Presentation_1.pdf]

Promoter hypomethylation and overexpression of TSTD1 mediate poor treatment response in breast cancer

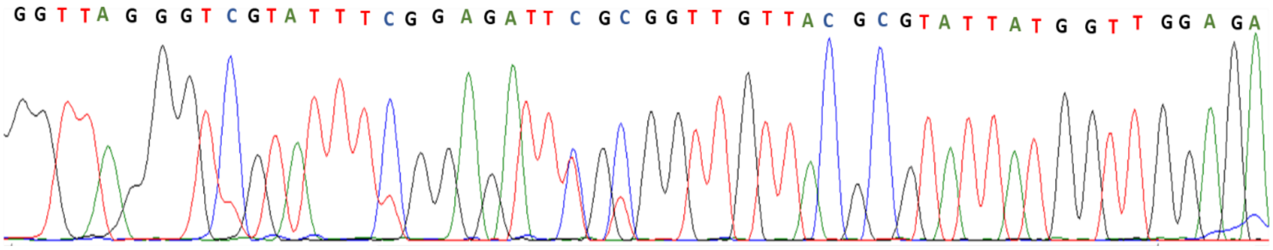

Figure S1. qPCR end-product of TSTD1 methylation analyzed by bisulfite sequencing.

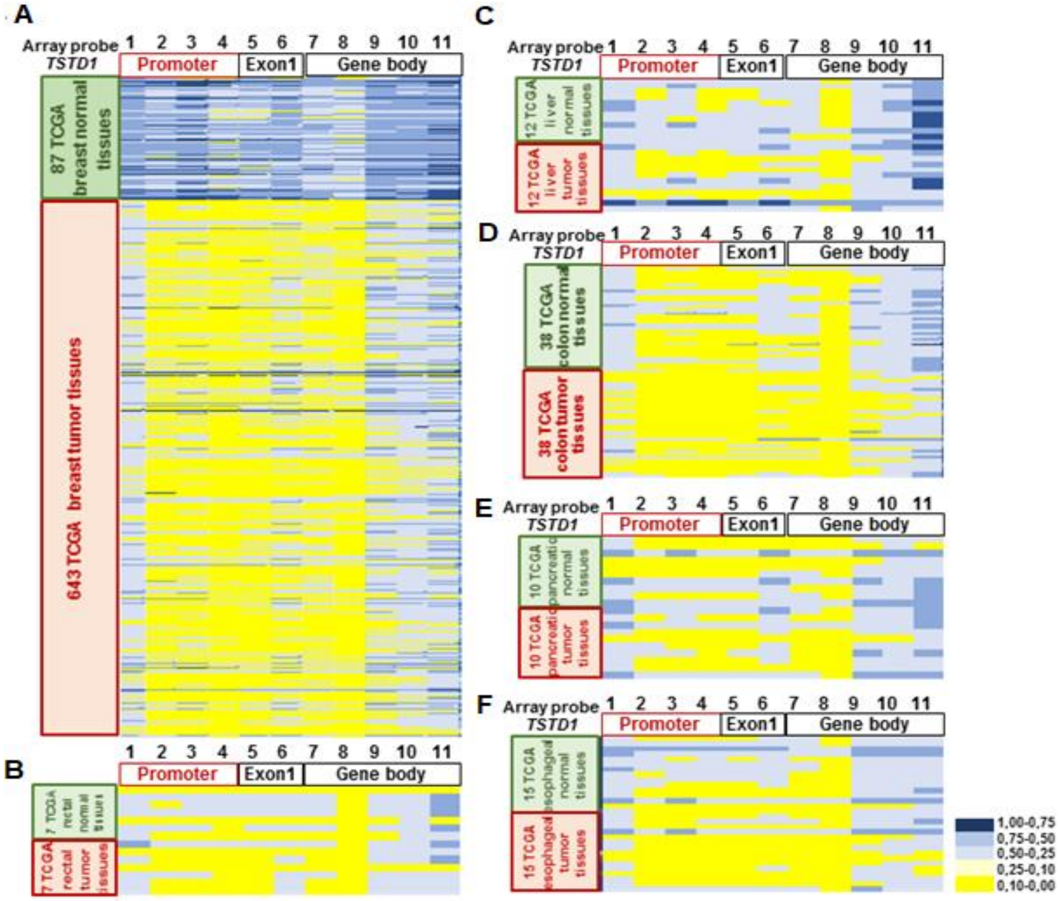

Figure S2. ***TSTD1* methylation heatmap.** *TSTD1* methylation pattern in **(A)** non-paired breast cancer, **(B)** paired pancreatic cancer, **(C)** paired liver cancer, **(D)** paired colon cancer, **(E)** paired esophageal cancer, and **(F)** paired rectal cancer. The methylation level belongs to 4 CpG sites in promoter regions −203, −50, −37 and −122 are designated 1, 2, 3 and 4 respectively. The CpG sites in exon 1 regions +24 and +69 are designated 5 and 6. The CpG sites in body regions +130, +232, +312, +477 and +647 are designated 7, 8, 9,10, and 11 respectively.

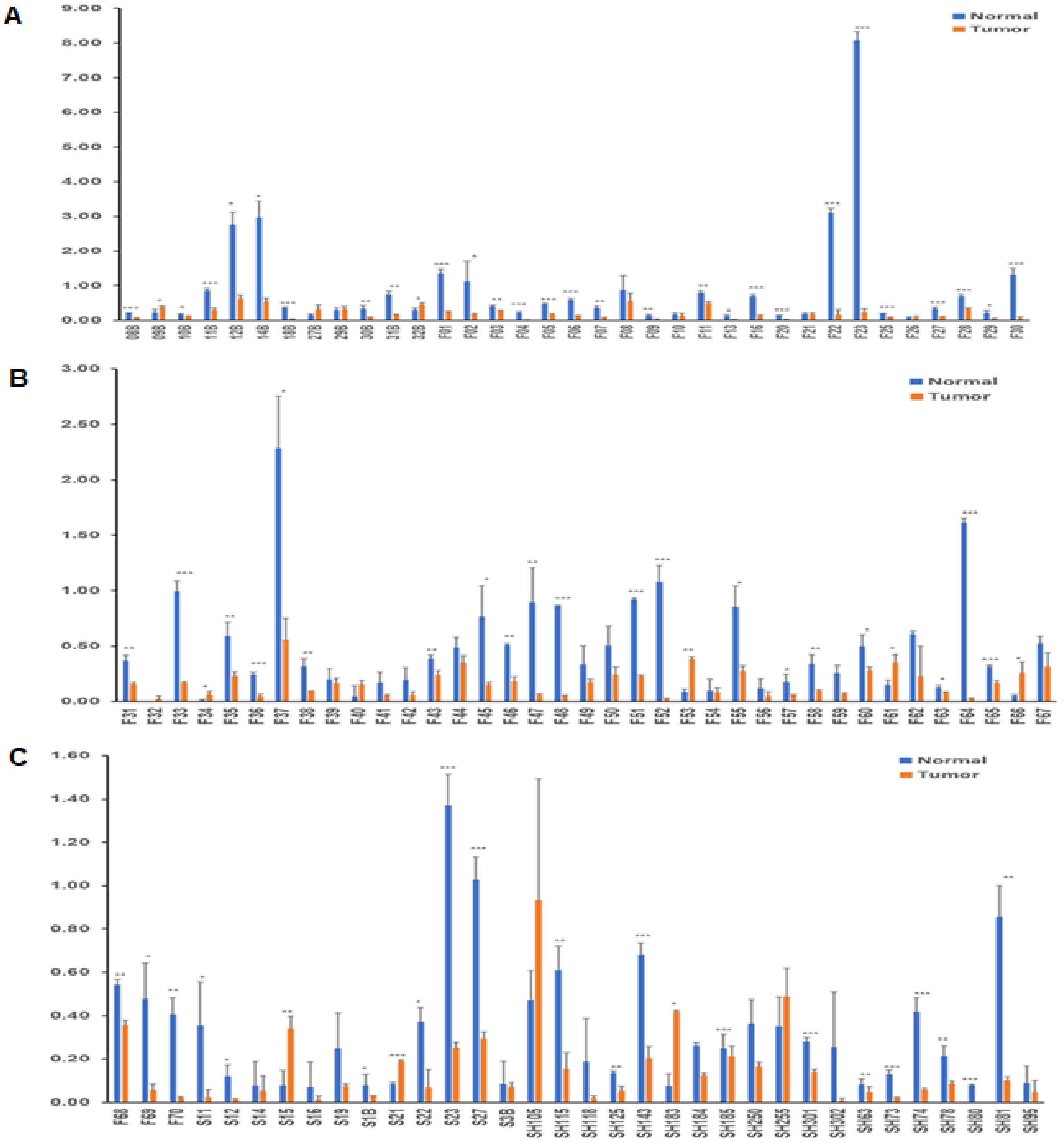

Figure S3. Methylation level of *TSTD1* in Taiwanese breast cancer patients. Tumor and adjacent tissue samples of 106 patients were obtained. ACTB was used as internal control. Experiments were performed triplicated (continued in next page). * $p \leq 0.05$, ** $p \leq 0.01$, *** $p \leq 0.001$.

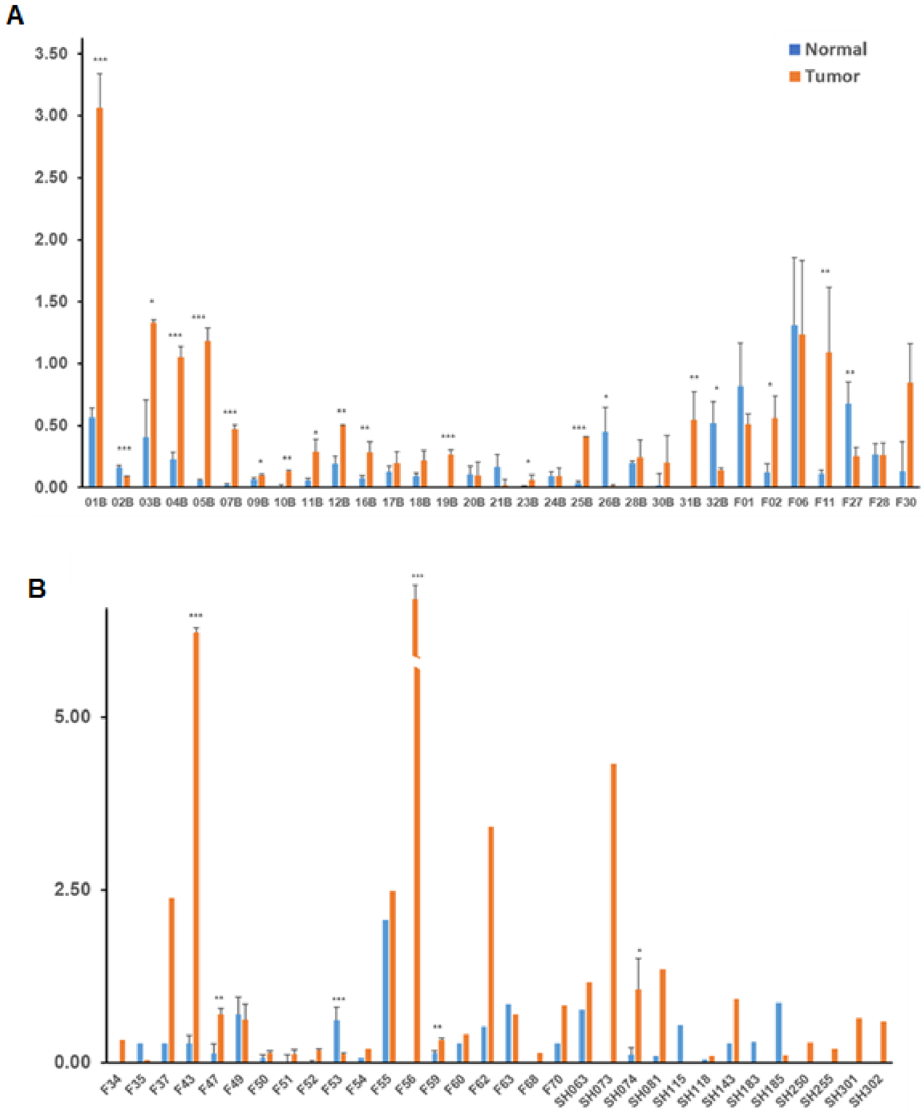

Figure S4. Expression level of TSTD1 in Taiwanese breast cancer patients. Tumor and adjacent tissue samples of 63 patients were obtained. GAPDH was used as internal control. Initially, experiments were performed in triplicate to control for variation (n = 40). Then, because of the lack of samples, experiments were performed once for each sample (n = 23). *, $p < 0.05$; **, $p < 0.01$; ***, $p < 0.001$.

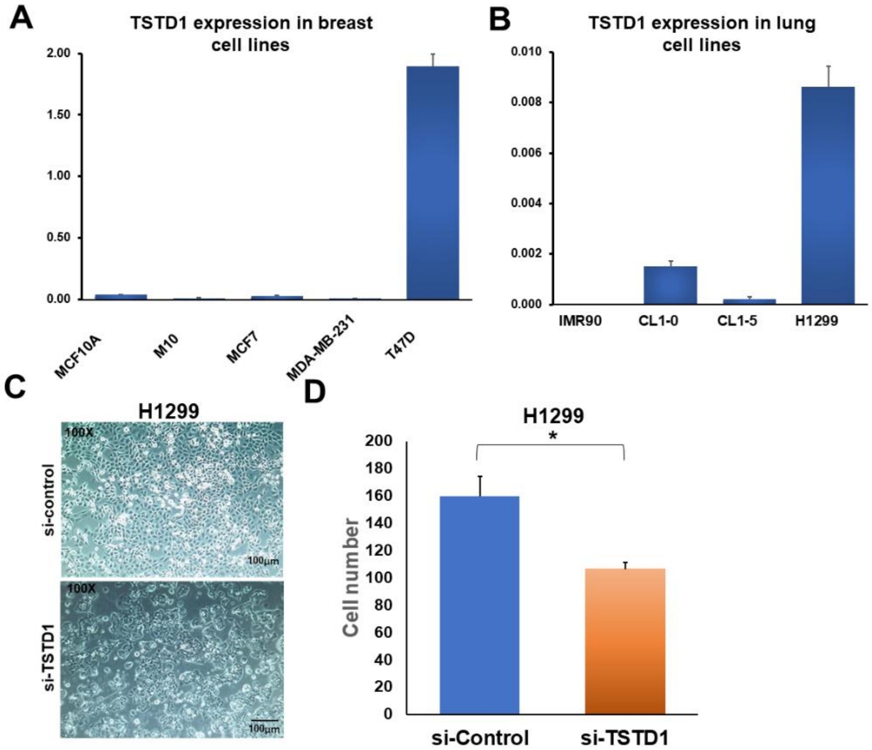

Figure S5. TSTD1 expression repressed the proliferation of cancer cell lines. GAPDH was used as internal control. TSTD1 expression was determined in **(A)** breast and **(B)** lung cell lines. **(C)** Images of H1299 lung cancer cell lines after transfection with si-TSTD1 **(D)** Bar chart shows the relative proliferation of H1299 cells using cell counting. The data are presented as the mean ± SD, * $p \leq 0.05$, ** $p \leq 0.01$, *** $p \leq 0.001$.

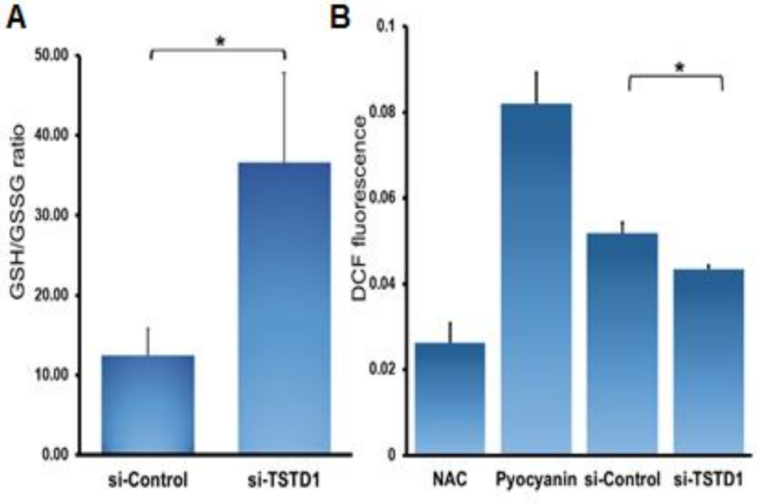

Figure S6. Knockdown of TSTD1 increased GSH/GSSG and reduced ROS. (A) GSH/GSSG ratio, **(B)** ROS levels in T47D cell line after knockdown of TSTD1. The data are presented as the means ± SD. * $p \leq 0.05$, ** $p \leq 0.01$, *** $p \leq 0.001$.

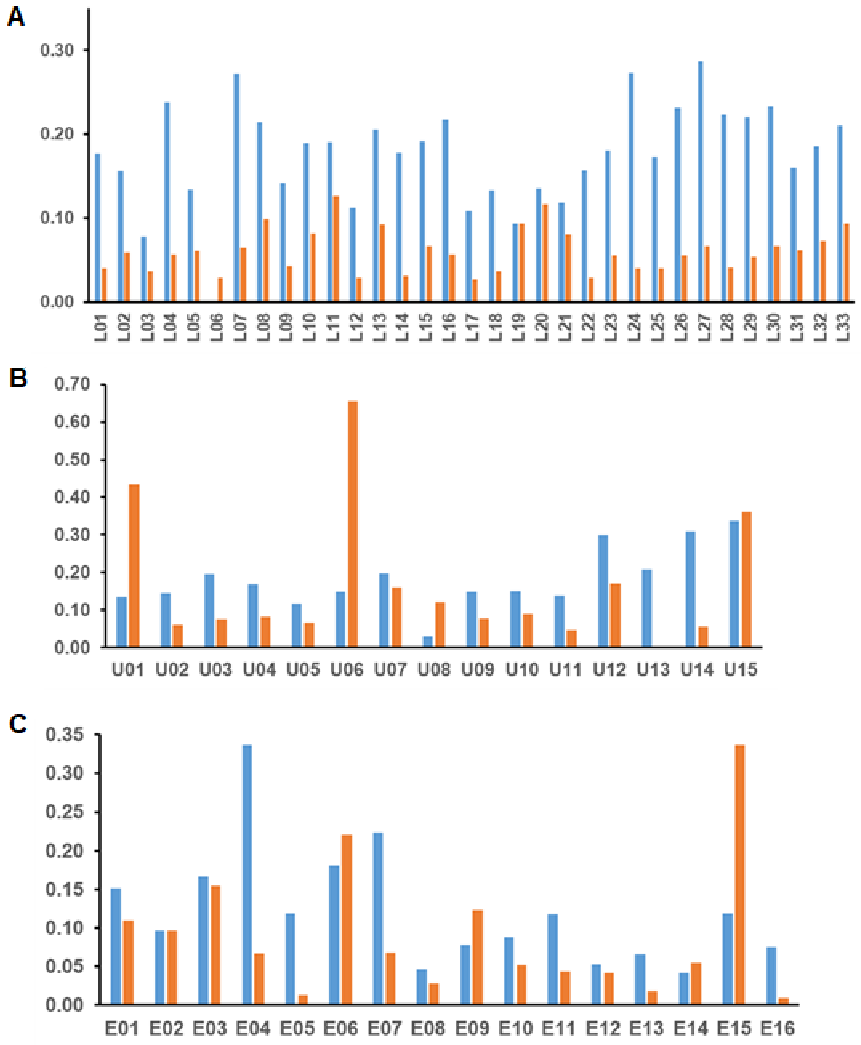

Figure S7. Methylation level of TSTD1 in Taiwanese lung, endometrial and esophageal cancer patients. Tumor and adjacent tissue samples of patients were obtained: **(A)** 33 pairs of lung cancer, **(B)** 15 pairs of endometrial, and **(C)** 16 pairs of esophageal cancer patients. ACTB was used as internal control.

.

Table S1. List of primer and probe sequences using for RT-qPCR and qMSP in present study.

Characteristic		Sequence
TSTD1-qMSP	Forward	GTTTTAGTTTTGGGAGGGAAC
	Reverse	CTCCAACCATAATACGCGTA
	Probe	TCTCCGAAATACGACCCTAACCCGC
TSTD1-RT-qPCR	Forward	CTCTTCGACGTGCGCTCT
	Reverse	CACTCTCCAACTCGGACACC
	Probe	#33
ACTB-qMSP	Forward	AACCAATAAAACCTACTCCTCCCTTAA
	Reverse	TGGTGATGGAGGAGGTTTAGTAAGT
	Probe	ACCACCACCCAACACACAATAACAAACACA
GAPDH-RT-qPCR	Forward	AGCCACATCGCTCAGACAC
	Reverse	GCCCAATACGACCAAATCC
	Probe	#60
ESR1-RT-qPCR	Forward	ATCCACCTGATGGCCAAG
	Reverse	GCTCCATGCCTTTGTTACTCA
	Probe	#17
ESR2-RT-qPCR	Forward	ATGATGGGGCTGATGTGG
	Reverse	TTCTACGCGCATTTCCCCTCA
	Probe	#50

Table S2. Significant relation between DNA hypomethylation and mRNA overexpression of *TSTD1* in Taiwanese and Western breast cancer patients[1].

	Total	Hypomethylation[2]	Non-Hypomethylation[3]	Person correlation
		**n (%)**	**n (%)**	
**TCGA**				
Down-Normal regulated[4]	22	10 (45.5%)	12 (54.5%)	-0.453 Sig.(2-tailed) ≤0.001***
Up-regulated[5]	50	44 (88.0%)	6 (12.0%)	
**Taiwanese**				
Down-Normal regulated	16	7 (43.8%)	9 (56.3%)	-0.300 Sig.(2-tailed) = 0.040*
Up-regulated	31	23 (74.2%)	8 (25.8%)	

*, $p < 0.05$; **, $p < 0.01$; ***, $p < 0.001$.

[1] These results were analyzed by the Person's correlation. *P* values with significance are shown as superscripts.

[2]*TSTD1* methylation was considered hypomethylation when the *TSTD1* methylation level was less than half in breast tumors compared to adjacent normal breast tissues.

[3]*TSTD1* methylation was considered Non- Hypomethylation when the *TSTD1* methylation level was less or equal than half in breast tumors compared to adjacent normal breast tissues.

[4]*TSTD1* was considered Down-Normal regulated when the *TSTD1* expression level was less or equal than one and a half in breast tumors compared to adjacent normal breast tissues.

[5]*TSTD1* was considered Up-regulated regulated when the *TSTD1* expression level was less than one and a half in breast tumors compared to adjacent normal breast tissues. Results were analyzed by Pearson's X^2 test.
